# Surgical Treatment of a Catheter-Induced Iatrogenic Dissection of the Right Coronary Artery following Cardiac Catheterization

**Published:** 2016-01-13

**Authors:** Panagiotis Artemiou, Stefan Lukacin, Peter Kirsch, Jan Ignac, Boris Bily, Alzbeta Tohatyova, Miroslava Bilecova-Rabajdova, Frantisek Sabol

**Affiliations:** 1 *University of P. J. Safarik, Medical Faculty, Department of Cardiovascular Surgery, Kosice, Slovakia.*; 2 *University of P. J. Safarik, Medical Faculty, Department of Cardiology, Kosice, Slovakia.*; 3 *University of P. J. Safarik, Medical Faculty, Children’s Hospital, Kosice, Slovakia.*; 4 *University of P. J. Safarik, Medical Faculty, Department of Clinical Biochemistry, Kosice, Slovakia.*

**Keywords:** *Cardiac catheterization*, *Dissection*, *Iatrogenic disease*

## Abstract

Iatrogenic dissections of the ascending aorta are an uncommon and severe complication during cardiac catheterization. A 68-year-old female patient underwent diagnostic cardiac catheterization due to non-ST-elevation myocardial infarction. During the procedure, a catheter-induced 360^° ^Class I dissection of the right coronary artery occurred. The patient developed severe bradycardia, which was treated with a temporary pacemaker. She underwent an emergency operation with ligation and a saphenous vein graft in the right coronary artery. The postoperative course was uneventful; and on postoperative day 6, she was discharged home.

## Introduction

Iatrogenic dissections of the ascending aorta are an uncommon and severe complication during heart catheterization. The incidence ranges between 0.02 and 0.08% in diagnostic procedures and between 0.07 and 0.6% during percutaneous coronary interventions (PCIs).^1^^-^^10^ The main mechanism involved is the retrograde extension of a coronary artery dissection into the aortic root.^11^ The clinical presentation and outcome of iatrogenic aortic dissections seem to differ from those of spontaneous dissections.^12^

In this report, we present a catheter-induced iatrogenic dissection of the right coronary artery following cardiac catheterization.

## Case Report

A 68-year-old woman with a history of arterial hypertension well controlled on medical treatment, hypercholesterolemia treated with simvastatin, and paroxysmal atrial fibrillation treated with amiodarone was admitted with unstable angina, elevated troponin levels, and ischemic electrocardiographic changes on leads V4-V6. Moreover, she suffered from hepatopathy and subclinical hypothyroidism and had previously undergone mastectomy due to breast cancer.

**Figure 1:**
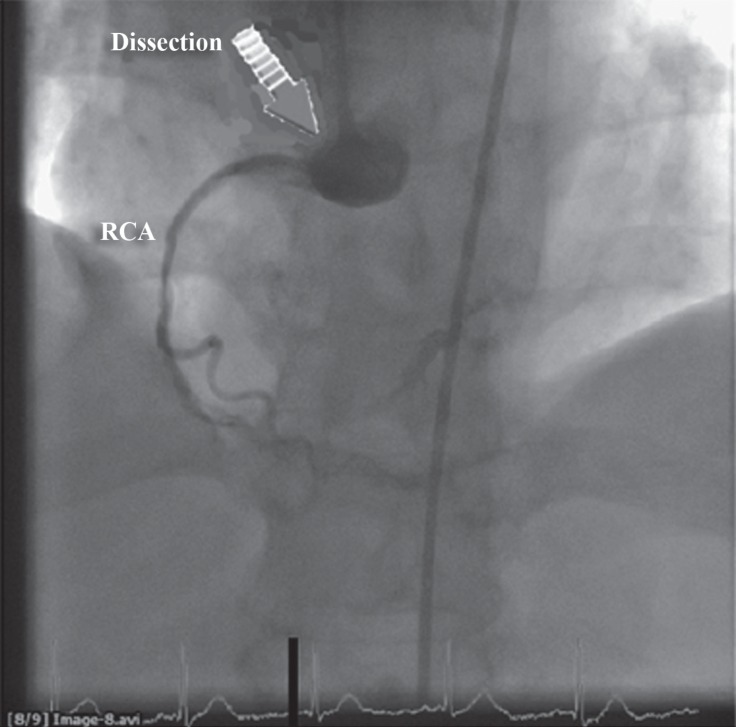
Coronary angiogram (left anterior oblique projection) – arrow showing the aortocoronary dissection of the right coronary artery (RCA)

The patient underwent diagnostic cardiac catheterization with normal coronary arteries. The left main coronary artery was selectively cannulated with a 6 F Judkins Left-4 guiding catheter and the right coronary artery was also selectively cannulated with a 6 F Judkins Right-4 guiding catheter (Medtronic, Inc., Minneapolis, Minnesota). The velocity of the contrast injection in both coronary arteries was 3 mL/s. In both arteries, the cannulation was uneventful without any complication. A second fluoroscopic injection into the right coronary artery demonstrated a 360^°^ dissection of the right coronary artery extending to the coronary cusp with a subtotal occlusion of the artery (Figure 1). 

**Figure 2:**
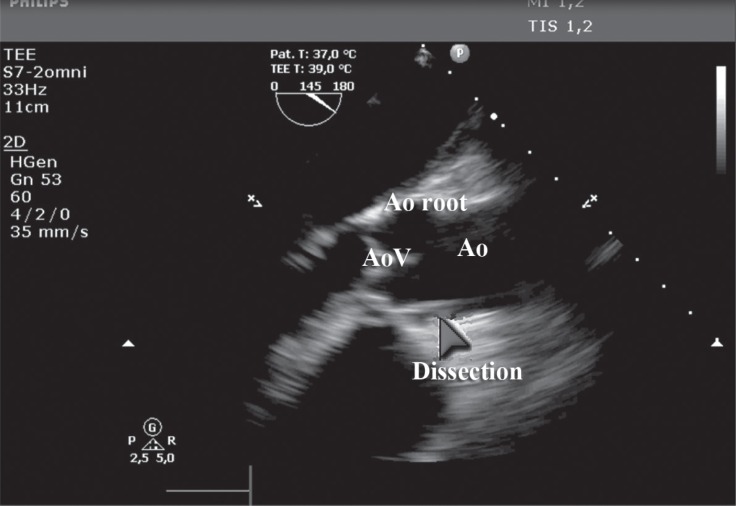
Transesophageal echocardiogram (long axis) – arrow showing the aortocoronary dissection

The patient developed severe bradycardia, which was treated with a temporary pacemaker. Emergency transthoracic echocardiography in the catheterization laboratory and later perioperative transesophageal echocardiography confirmed the dissection and demonstrated acute akinesis of the diaphragmatic myocardial wall and the interventricular septum with moderate left ventricular dysfunction (Figure 2).

Ao, Aorta; AoV, Aortic valve

**Figure 3:**
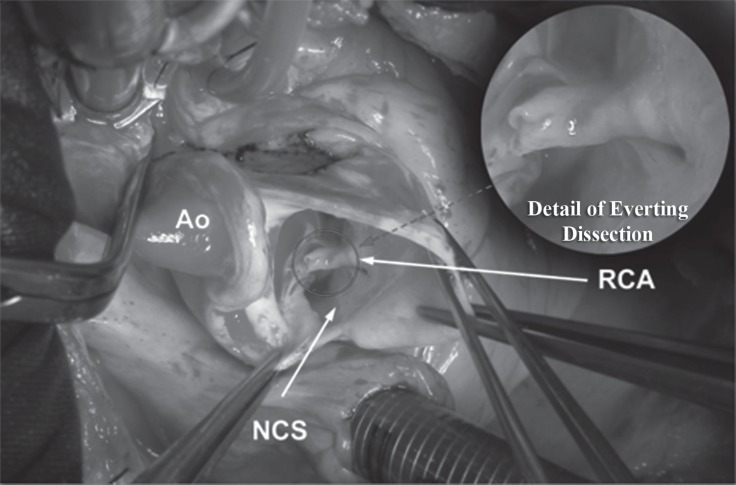
Intraoperative finding - arrow showing the everting aortocoronary dissection

**Figure 4:**
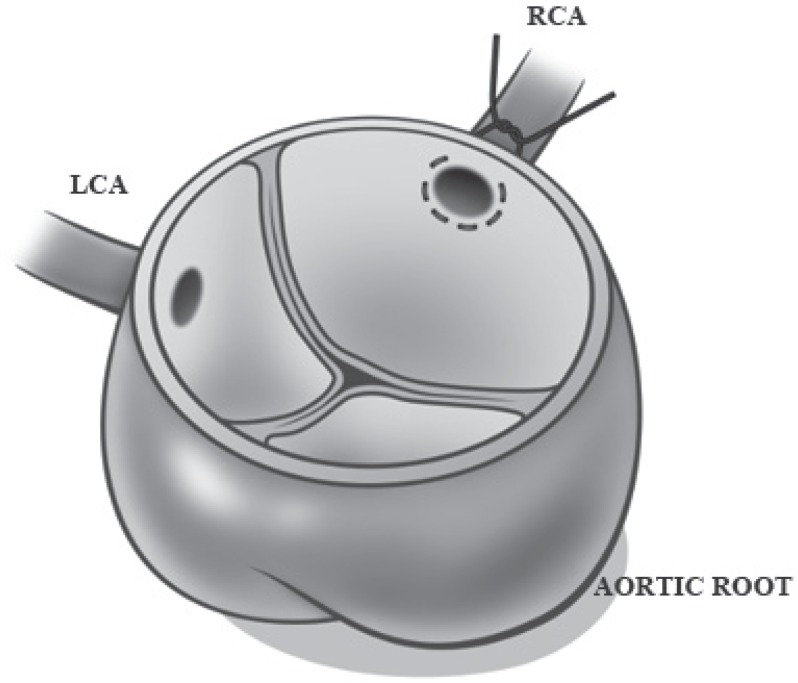
Intraoperative procedure – drawing showing ligation of the right coronary artery at its origin

LCA, Left coronary artery; RCA, Right coronary artery

The patient underwent an emergency operation through a median sternotomy with ligation of the right coronary artery at its origin with a saphenous bypass vein graft. The aorta was opened, and no aortic wall dissection was seen. The operative finding showed a 360° dissection of the right coronary artery with the dissecting flap everting into the aortic lumen (Figure 3). The dissection on the right coronary artery was extended distally to the bifurcation of the artery. The venous graft was placed distally before the bifurcation of the artery, and the lumen of the artery at the anastomotic site was dissected. The operative procedure is shown in Figure 4.

The postoperative course was uneventful; and on postoperative day 6, she was discharged home in stable condition.

Ao, Aorta; RCA, Right coronary artery; NCS, Non-coronary sinus

## Discussion

Catheter-induced dissection with retrograde extension to the aortic root, a feared complication of cardiac catheterization, is rare and has been estimated to occur in approximately 0.02 to 0.08% of diagnostic catheterization and 0.07 to 0.6% of PCIs, but the overall incidence of catheter-induced dissections remains unknown.^10^^, ^^13^

In our patient, aortocoronary dissection was the complication of a catheter-induced dissection of the right coronary artery. This finding is consistent with those in the literature. Goldstein et al.^14^ reported that 89% of dissections involve the right side and 11% the left side. The media of the left coronary artery contains more circulatory and spiral smooth muscle cells than does the right coronary artery. These cells are arranged in concentric layers with abundant elastic fibers, which may explain the higher resistance of the left main coronary artery to retrograde dissections.^15^

A proposal was made by Dunning et al.^11^ for a classification system based upon the extent of aortic involvement. Class I: The contrast staining involves the coronary cusp; Class II: The contrast extends up the aortic wall < 40 mm; and Class III: The contrast extends > 40 mm up the aortic wall. In their series, patients with Class III dissections had poor outcomes. This classification may be useful for risk stratification. According to this classification, our patient had a Class I dissection with a favorable outcome.

The risk factors for aortocoronary dissections include hypertension, older age, extensive atherosclerosis and underlying structural weakness of the media (cystic medial necrosis),^16^ use of Amplatz-shaped catheters, and catheterization for acute myocardial infraction.^11^ Other possible risk factors include catheter manipulation and deep intubation of the catheter within the coronary artery,^17^ vigorous contrast injection and vigorous deep inspiration,^18^^, ^^19^ and finally a variant anatomy of the coronary ostia.^20^

The management of the catheter-induced coronary artery dissection depends on the patency of the distal vessel and the extent of the propagation of the dissection. If there is a compromise of the distal artery bed, such as the acute closure of the artery, urgent revascularization is mandated to prevent the infarction of that myocardial area. This may be achieved via PCI or coronary artery bypass graft surgery (CABG). Our patient presented with acute hemodynamic instability and a third-degree atrioventricular block, so the decision for urgent CABG was made. In the literature, there have also been reports of successful outcomes with CABG.^18^^, ^^21^ In contrast in some cases, the treatment of choice regarding aortocoronary dissections has involved immediate stenting limited to the coronary artery and its ostium to seal the entry point of the dissection without intervention in the aortic extension of the dissection.^15^^, ^^17^^, ^^22^^-^^24^ Also, localized aortocoronary dissections that appear stable throughout the procedure can probably be managed conservatively.^13^

Surgical intervention in the aorta via either aortic replacement or glue aortoplasty^25^^, ^^26^ along with coronary bypass can be considered depending on the finding of immediate coronary stenting,^15^ aortic extension,^11^ or progression^27^ of the dissection and the clinical condition of the patient such as hemodynamic instability.

Invasive and noninvasive imaging methods have been used to visualize and follow aortocoronary dissections induced in the catheterization laboratory. In our case report, catheter coronary angiography and aortography was utilized for an immediate diagnosis of the coronary artery dissection and its aortic extension. Also, transesophageal echocardiography was employed in the catheterization laboratory for an accurate definition of the dissection and evaluation of the aortic valve function. Intravascular ultrasound also has been used to identify the entry point of the dissection.^28^ Moreover, computer tomography angiography has been drawn upon as a noninvasive modality for a precise diagnosis and follow-up of the aortic extension of a coronary artery dissection induced in the catheterization laboratory.^29^ In some cases, however, magnetic resonance imaging has been used for the follow-up of the aortic extension of the dissection.^30^

## Conclusion

Although in Class I dissections the surgical approach is not the first choice, in our case report we used it for the following reasons. The patient presented with acute hemodynamic instability alongside arterial hypotension and third-degree atrioventricular block and - in addition - the echocardiographic picture was not clear enough to determine the class of the dissection. Indeed, the definite diagnosis of the dissection was determined intraoperatively. Given the intraoperative finding, CABG was a more appropriate treatment than was PCI. In conclusion, the seriousness of our patient’s clinical condition and echocardiographic findings constituted the reasons for us to proceed with a surgical approach.

## Notes:

***This paper should be cited as:*** Artemiou P, Lukacin S, Kirsch P, Ignac J, Bily B, Tohatyova A, Bilecova-Rabajdova M, Sabol F. Surgical Treatment of a Catheter-Induced Iatrogenic Dissection of the Right Coronary Artery following Cardiac Catheterization. J Teh Univ Heart Ctr 2016;11(1):30-33.
